# Feasibility of Standardized Human Milk Collection in Neonatal Care Units

**DOI:** 10.1038/s41598-019-50560-y

**Published:** 2019-10-04

**Authors:** Laura Galante, Mark H. Vickers, Amber M. Milan, Clare M. Reynolds, Tanith Alexander, Frank H. Bloomfield, Shikha Pundir

**Affiliations:** 1 The Liggins Institute, The University of Auckland, Auckland, New Zealand; 2 Neonatal Unit, Kidz First, Middlemore Hospital, Auckland, New Zealand; 3 Newborn Services, Auckland City Hospital, Auckland, New Zealand

**Keywords:** Medical research, Health care

## Abstract

Research in human lactation is a growing field. However, difficulties in studying human milk originate from the dynamicity of its composition. Using standardized collection protocols is mandatory to minimize variation and warrant comparability of findings across different studies. Yet, information on the feasibility of collecting human milk with standardized procedures, especially in neonatal units, are lacking. The present study aims to report on the feasibility and difficulties to collect human milk according to a standardized protocol, during early lactation from women who gave birth to preterm infants. Human milk was collected from 129 mothers of moderate- to late-preterm infants according to two variations of a standard protocol which differed for number of collection time-points. Collection rates and adherence to the sampling protocol were evaluated together with reason for missed collection. Collection of ≥1 sample was successful for 80% of the mothers. However adherence to the standard protocol was overall low (36% and 27%). Collection rates were different between the two protocol variations (73% against 92%, p ≤ 0.001). Amongst the reason for missed collection, low milk supply was the most recurrent (40%). Our findings show that while collecting human milk in neonatal units is achievable, obtaining standard and comparable samples results challenging.

## Introduction

Logistical difficulties around the examination of the biochemical composition of human milk (HM) are well established^1^. HM composition is extremely dynamic and changes in relation to several factors, including maternal diet and metabolic conditions^2,3^, lactation stage^4^, length and frequency of the feeds^5^ and circadian rhythms^6,7^. For this reason, the time and mode of collection has an impact on the outcome of compositional analysis^8^.

The impact of collection method on HM analysis was first described in 1982^9^. Since then, few studies have investigated the effect of different collection methods (hand-expression versus breast pump-expression), storage containers (pyrex versus polypropylene), storage temperature and time to laboratory analysis, suggesting that expressed volumes of HM are dependent upon the specific collection method and that storage containers and sample storage conditions impact HM composition^10,11^. As HM research expands, concerns around accurate, reproducible and comparable results from HM compositional studies have highlighted a need for the careful evaluation of the most suitable and reliable collection method(s)^1,12,13^.

At present, different collection methods are used across different studies and some studies also report inconsistency of collection methods within the same cohort. This often consists of variable and unspecified collection timeframes, mixed hand- and pump- expression and\or use of mixed collection instruments^13–16^. However, despite the recognition of the importance of standardizing HM collection methods, in many cases it is logistically and sometimes ethically challenging to do so, given the nature of the sample. For instance, some variables that impact the composition of HM, such as the time at which a mother expresses or breastfeeds, depend upon the infant and on the mother’s lifestyle and, as such, are often not adaptable to the requirements of the researcher.

The difficulty in standardizing collection increases in settings such as neonatal care units (NCU, including neonatal intensive care units or special care baby units) where many factors, especially those associated with preterm birth, can obstruct or delay lactogenesis^17^. In such environments, any HM produced by mothers is extremely important for the infants^18^, as it reduces morbidity and mortality in preterm and very low birthweight infants^19,20^. The beneficial effect of HM on complications faced by preterm infants, such as necrotizing enterocolitis^21^ and enteral feed intolerance^22^ and on neurodevelopmental outcomes^23^ is well established. However, the mechanisms of action that lead to most of these benefits remain poorly defined. Evidence shows that HM produced from mothers of preterm infants is different compared to HM produced from term mothers, having higher energy density and increased macronutrient^24^ and immune factor profiles^25^. As a result, the impact of mothers’ own milk composition, as opposed to any other milk or nutrition that preterm infants may receive, potentially affects the postnatal outcome of the infant^26^. For this reason, further research on HM sourced from mothers of preterm infants is essential to enable a better understanding of how HM components act and in order to assess the nutritional intake that preterm infants receive. However, no previous study has reported on the feasibility to standardize HM collection after preterm birth. The present study therefore reports our experience and barriers in trying to standardize HM sample collection in the NCU from mothers of moderate- to late-preterm infants, soon after birth.

## Methods

### Study design and population

The present research was designed to evaluate the adherence to standard sampling procedures for HM collection in the NCUs of maternity hospitals in Auckland, New Zealand. For this purpose, data around HM sample collection, including compliance to the standard collection protocol, were examined from mothers of moderate- to late-preterm infants who were recruited into the DIAMOND trial^27^ between July 2017 and August 2018 from four different hospitals across Auckland. Only mothers who consented to give at least one HM sample in the NCU were included in the present study. Ethical approval for the DIAMOND study and any related analysis was given by the New Zealand Health and Disability Ethics Committee (16/NTA/90). The participating sites and respective District Health Boards are Auckland City Hospital, Auckland District Health Board, Middlemore Hospital, Counties Manukau Health, North Shore Hospital and Waitakere Hospital, Waitemata District Health Board. The locality approvals were provided by each District Health Board’s Research Review Committee. Written, informed, consent was obtained from each participant. All methods were performed in accordance with the relevant guidelines and regulations.

### HM and data collection

HM was collected according to two variations of a standardized protocol. Variation A of the protocol was used to collect HM from July 2017 to Jan 2018 (N = 40 mothers). This required HM collection at days 3, 5 and 10 after birth and was later changed into Variation B (N = 62) which entailed collection only at day 5 and 10 after birth. In order to standardize collection, mothers were asked to collect the sample exclusively from the right breast between 10 am and 12 pm, 2–3 hours after they had emptied the same breast during the last expression or breastfeed. Nonetheless, collection time-points had to be made flexible to accommodate parents requirements. For this reason we allowed for a 1–2 days window before/after the exact collection day so that day 3 samples could be collected either on day 3 o 4 after birth (D3 = day 3 + 1), day 5 samples could be collected either on day 5, 6 or 7 (D5 = day 5 + 2) and day 10 samples could be collected either on day 8, 9, 10, 11 and 12 after birth (D10 = day 10 ± 2). For consistency, when a day 3 sample was collected on day 4, the following collection was also shifted by 1or 2 days. Research staff reminded mothers of the collection timing and of emptying the right breast 2–3 hours before coming to the hospital on the day before and on the morning of collection. During collection, mothers were asked to completely empty the breast with the use of a hospital-grade breast-pump (Medela, Switzerland), into disposable sterile bottles (Medela, Switzerland). The volume of the total expression was recorded for all mothers that attempted samples collection, together with time of collection and last expression from the right breast in order to verify compliance with the protocol. Expression frequency during the 24 hours prior to sample was also noted. From the total expressed volume, 2 ml were taken with a sterile enteral syringe (Medicina, United Kingdom) and aliquoted into low protein binding microtubes (Eppendorf, Germany). The remaining HM was returned to mothers to feed their infants. Although mothers had previously consented to provide a HM sample for the study, prior to each time-point the research team verified with the clinical staff that mother’s HM supply was enough to feed their infant and provide the sample. If mothers expressed less HM than required to feed their infant, the sample for the study was not collected. In any instance where a sample collection was not viable, the reason for the missed collection was recorded.

Maternal demographic information (i.e. including education and ethnicity) was collected at the time of recruitment, while clinical information (i.e. gestational diabetes, prenatal steroid treatment and mode of birth) were obtained from hospital records. Mothers were asked to complete two questionnaires around day 10 after birth to assess postnatal depression (Edinburgh Postnatal Depression Scale, EPDS) and perceived level of stress (Perceived Stress Scale, PSS). This information was used to evaluate any relationship between sample collection and maternal characteristics.

### Statistical analyses

Differences in frequencies for collection and adherence to protocol were analysed across groups using Fisher’s exact test, due to the limited number of frequencies in certain groups. Fisher’s exact test was also used to analyze the distribution of reasons around missed sample collections. Because expressed volumes of HM on the day 3 collection resulted skewed when tested for normality with the Shapiro-Wilk test, the comparison of HM volumes across groups were performed using Mann-Whitney test when comparing two groups or Kruskal-Wallis when comparing more than two groups. Associations between volumes of expressed HM, stress scores and expression frequency in the 24 hours prior to sample collection were analyzed with linear regression. All reported statistical results are two-tailed and all statistical analyses were performed using IBM SPSS Statistics (version 25).

## Results

### Eligibility

**Figure 1:**
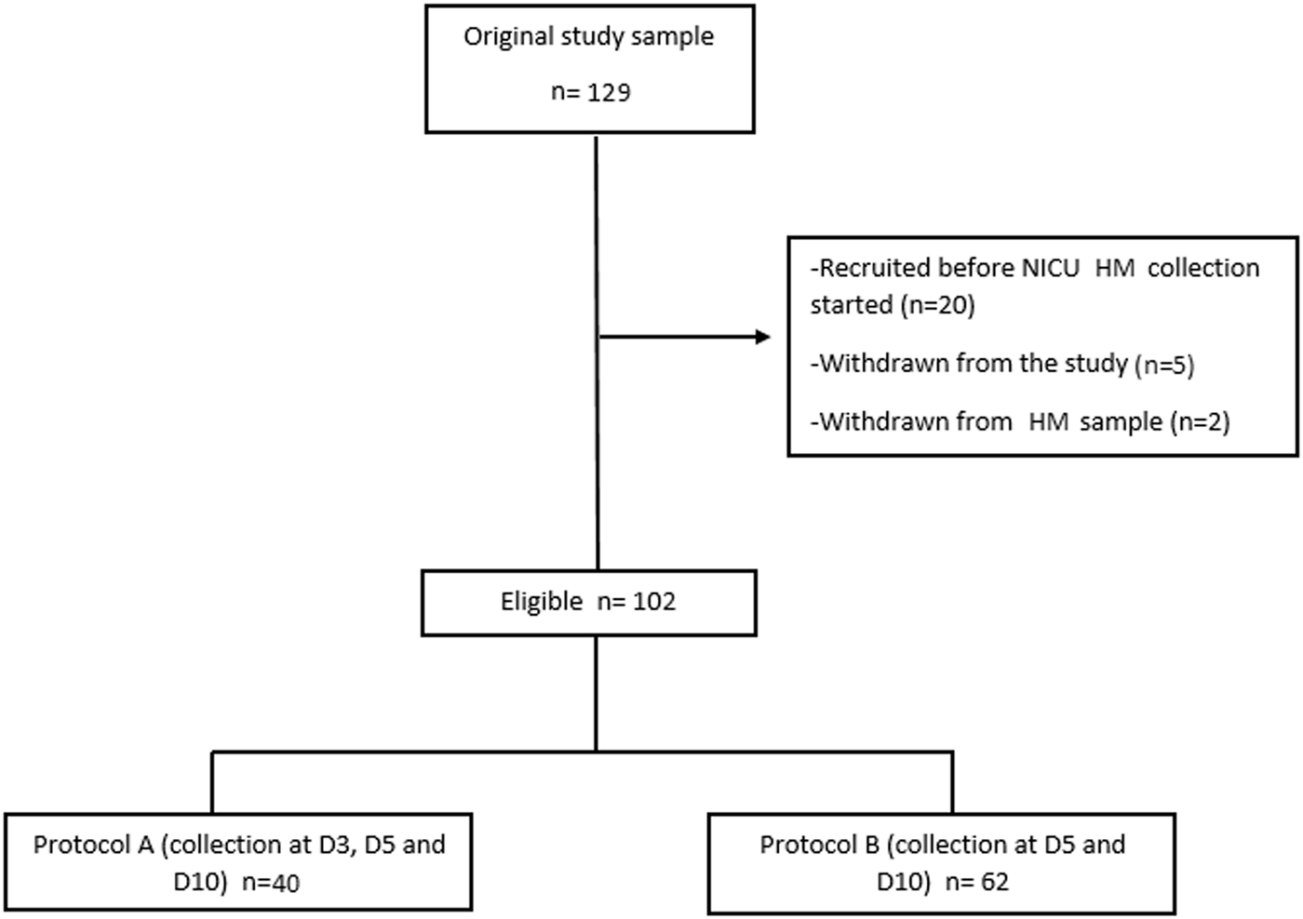
Flow chart of the included mothers.

Of the 129 mothers that were recruited in the DIAMOND study between July 2017 and August 2018, 20 mothers had been enrolled in the study prior to the start of HM collection in the NCU, five mothers withdrew their infants from the study and two mothers withdrew from HM collection. Thus 80% (N = 102) of the targeted population was included in the final analysis (Fig. 1).

### Population characteristics and variance across NCUs

Maternal and infant characteristics are summarised in Table 1. Asian and European ethnicity were the most represented, accounting for 71% of the total study population. Overall, 82% of the total number of HM samples were collected. No difference was observed in collection frequencies (collected versus missed) and adherence to protocol at each time-point across the four hospitals (data not shown). The fourth unit (Waitakere Hospital) started recruiting for the study after the sampling protocol was changed from A to B and for this reason it did not have any day 3 (D3) collection. Furthermore, because this unit started recruiting much later than the previous three, it displays a smaller number of mothers that enrolled in the study compared to all the other units.

**Table 1:** Maternal characteristics (N = 102) and association with sample collection.

Maternal and Infant characteristics	Total Mothers	Total sample in D3	Total sample	Samples collected D3 N (%)^a^	Samples collected D5 N (%)^a^	Samples collected D10 N (%)^a^	Total collected N (%)^a^
Maternal age (Mean ± S.D.)	31 ± 6						
**Maternal ethnicity**							
European	39	17	95	10 (59)	35 (90)	37 (95)	82 (91)
Maori	8	3	19	1 (33)	7 (88)	6 (75)	14 (74)
Asian	33	10	76	3 (30)	30 (91)	30 (91)	63 (83)
Pacific Islands	16	6	38	4 (67)	15 (93)	10 (63)	29 (76)
Other	6	4	16	1 (25)	6 (100)	6 (100)	13 (81)
**Maternal education**							
No education	1	0	2	—	1 (100)	1 (100)	2 (100)
Lower secondary school	9	4	22	1 (25)	9 (100)	8 (89)	18 (82)
Upper secondary school	20	12	52	7 (58)	18 (90)	15 (75)	40 (77)
Post-secondary, non-tertiary or short cycle of tertiary	14	2	30	1 (50)	11 (77)	11 (79)	23 (77)
University	57	21	135	9 (43)	53 (93)	53 (93)	115 (85)
Other	1	1	3	1 (100)	1 (100)	1 (100)	3 (100)
**Maternal gestational diabetes mellitus (GDM)**							
No	82	30	194	15 (50)	76 (93)	72 (88)	163 (84)
Yes	20	10	50	4 (40)	17 (85)	17 (85)	38 (76)
**Infant sex**							
Male	55	19	129	6 (32)	53 (96)	48 (88)	107 (83)
Female	34	14	82	8 (57)	30 (88)	30 (88)	68 (83)
Male twins	7	4	18	3 (75)	4 (57)	5 (71)	12 (67)
Female twins	4	1	9	1 (100)	4 (100)	4 (100)	9 (100)
Different sex twins	2	2	6	1 (50)	2 (100)	2 (100)	5 (83)
**Birth mode**							
Vaginal	42	16	100	6 (38)	41 (98)	35 (83)	82 (82)
C-section	59	23	141	12 (52)	51 (86)	54 (92)	117 (83)
Missing	1	1	3	1 (100)	1 (100)	0 (0)	2 (67)
**Antenatal steroids**							
None	20	9	49	4 (44)	19 (95)	13 (65)	36 (73)
Incomplete (less than 24 h before birth)	22	5	49	3 (60)	20 (91)	22 (100)	45 (92)
Complete (more than 24 h before and less than 7 days before birth)	57	24	138	11 (46)	51 (90)	51 (89)	113 (82)
More than 7 days before birth	1	0	2	—	1 (100)	1 (100)	2 (100)
Multiple courses	2	2	6	1 (50)	2 (100)	2 (100)	5 (83)
**Gestational age (weeks)**							
32	26	12	64	7 (58)	21 (81)	24 (92)	52 (81)
33	23	6	52	2 (33)	23 (100)	22 (96)	47 (90)
34	32	12	76	7 (58)	29 (91)	27 (84)	63 (82)
35	21	10	52	3 (30)	20 (95)	16 (76)	39 (75)
Total	102	40	244	19 (48)	93 (91)	89 (87)	201 (82)

Percentages for day 3 (D3) sample were calculated based on the number of mothers included in variation A of the protocol (N = 40). Day 5 (D5) and day 10 (D10) percentages were calculated on the overall number of mothers considered for the present study (N = 102). ^a^Calculated based on total sample per time-point per subgroup (row).

### Feasibility of HM collection

**Figure 2:**
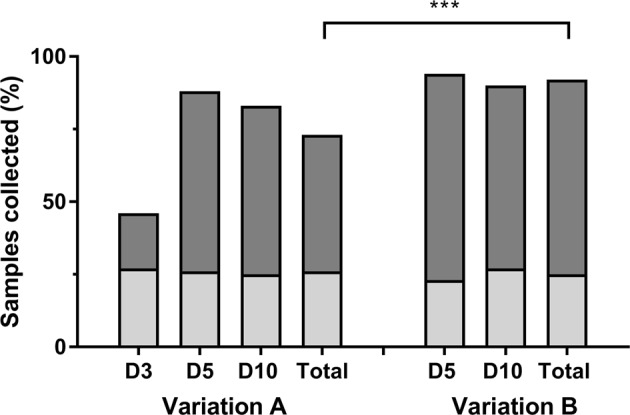
Collection rates ad adherence to standard sampling procedures (%) for the two protocol variations (A and B) utilised for HM collection during the DIAMOND trial. A (N = 40 mothers) entailed collection at day 3, 5 and 10 after birth, while B (N = 62 mothers) only entailed collection at day 5 and 10 after birth. The clearer portion of the bar indicates the amount of samples collected according to the standardized protocol. ***p ≤ 0.0001 between % of total samples collected in A and % of total samples collected in B. No difference was observed in relation to adherence to the standard protocol between A and B.

As shown in Fig. 2, the collection between variation A and B of the protocol was different, 73% and 92% respectively, (p ≤ 0.001). However, no difference was detected between collection at day 5 and 10 for the two variations. Only 31% of the samples were collected according to the protocol (between 10 am and 12 pm, 2–3 hours after the last expression, Fig. 2). The rest of the samples were collected out of the specified timing (49%) and/or mothers emptied the breast less/more than 2–3 hours before collection (56%). All mothers used the right breast. No difference was observed in terms of compliance to the standardized protocol across variation A and B (Fig. 2) and across the four sites (not shown).

### Barriers to HM collection and HM volumes

**Figure 3:**
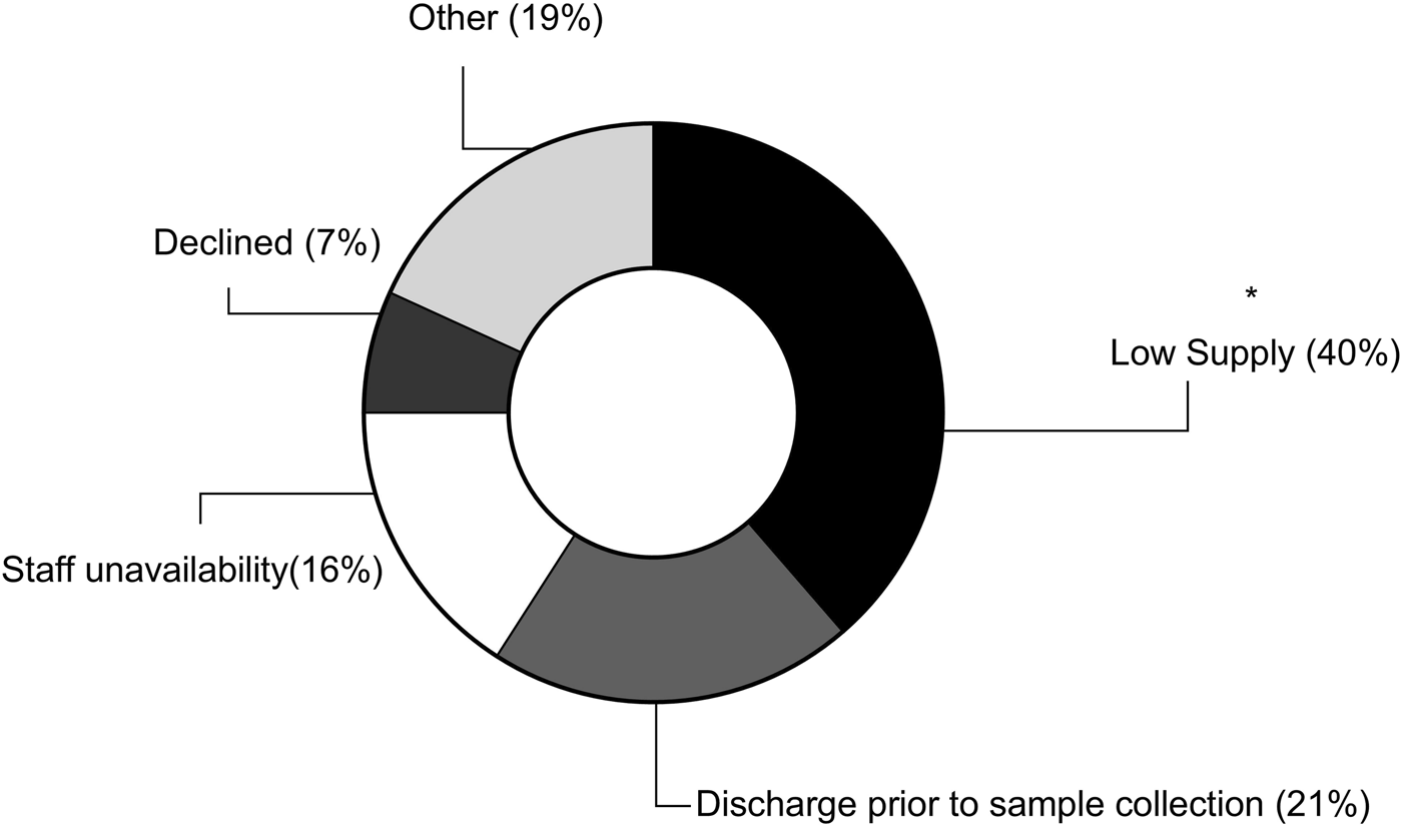
Summary of the most relevant reasons for missed HM collection. *p ≤ 0.01 when compared to the expected distribution of reasons for missed collection according to chi-squared analysis. Low supply: the mother did not produce enough HM to feed the infant and provide a sample. Discharge prior to sample collection: the infant was discharged from the hospital and sample could not be taken. However mothers had already provided at least one milk samples at other time-points. Declined: the mother did not feel comfortable donating a HM sample at a specific timepoint. However they did provide at least one milk sample at a different time-point. Staff unavailability: lack of clinical trained staff to perform sample collection. Others: minor reasons (≤2 cases) including mother not visiting the hospital, mother unwell, human error and failing to record reason for missed sample.

Reasons underlying missed sample collections were recurrent and, as shown in Fig. 3, were grouped into four categories plus one (others), which included the less frequent motivations. Low HM supply emerged as the predominant limiting factor (Fig. 3), especially on day 3. At this time-point the number of samples missed due to maternal low milk supply was significantly higher (p = 0.007) than samples missed due to any other reason.

Volumes of HM expressed by mothers did not differ across the collection sites. As expected, HM volume, assessed in mothers who provided samples across all time-points (n = 8 per group) increased (p < 0.05) from day 3 to day 10 (D3 = 28.75 ± 6.9 ml, D5 = 61.87 ± 11.21, D10 = 73.25 ± 12.4, Mean ± SEM). Volumes were also analysed against the maternal characteristics shown in Table 1. No significant difference was found between volumes of expressed HM at different time-points across any of these maternal characteristics, except for ethnicity (p = 0.004 on D5) and education (p = 0.018 on D5). In these cases, Pacific Island mothers showed a significantly higher milk supply on the day 5 collection (80 ± 21 ml, Mean ± SEM) compared to Asians (29 ± 5 ml, Mean ± SEM, p = 0.016) and mothers with a university degree displayed significantly lower HM volumes on D5 (32 ± 3.8 ml, Mean ± SEM) compared to mothers with a upper secondary school title (70 ± 15 ml, Mean ± SEM, p = 0.007).

EPDS (6 ± 5.5, Mode ± IQR) and PSS (15 ± 9, Mode ± IQR) scores showed that most mothers were moderately stressed but overly not depressed. However the two scores showed large variance with some mothers experiencing possible postnatal depression and high stress. Nonetheless both stress scores did not show any interaction with expressed HM volumes (D3 volume p = 0.514, D5 volume p = 0.511, D10 volume p = 0.330).

Data around expression frequency during the 24 hours prior each sampling showed that majority of the mothers expressed every three hours (Mode ± IQR for D3, D5 and D10 respectively 8 ± 4, 8 ± 2, 8 ± 2) as suggested by local guidelines^28^. Also in this instance, we found no association with expressed HM volumes at all the three time-points (D3 volume p = 0.252, D5 volume p = 0.615, D10 volume p = 0.615).

## Discussion

The purpose of the present study was to report on the feasibility to HM collection from mothers of preterm infants in NCUs and to provide an insight around possible implementation of standardized collection protocols.

According to our results the collection of HM samples in the NCU is overall achievable as only 43 (18%) samples were missed over the total 244 samples that could have been collected. However, the study shows an underlying difficulty in collecting samples according to standardized procedures, with the majority (69%) of the samples not collected in accordance with the protocol. Ideally, standardized HM collection should occur from a single breast, at the same time and within the same time-interval from previous feed/expression for all participants^29^. The importance of standardizing HM collection arises from the high variability that HM composition shows across different mothers and within the same mother^30^. In such situations, standardizing the collection of HM samples means ascertaining that their composition will be comparable at the time of analysis. Thus, while collection of HM should not constitute an inconvenience for mothers who agree to it, it is crucial for research purposes to try to optimize such collection methods. Interestingly, 59% of the samples collected on the day 3 time-point were collected according to the standard protocol, against only 27% and 29% respectively for the day 5 and day 10 samples over the 2 protocols. This was most likely due to the fact that mothers would still be in hospital patients at this time, and therefore have more opportunity to attend the right collection timing. In hospital mothers would also have had more access to breast-pumps for expression prior to collection and they would have followed three-hourly expression routine recommended by the clinical staff.

Collection rates between A and B showed to be different, but only due to the collection at day 3, where most samples were missed. This confirms that HM collection can be especially problematic during the first few days after birth, when its production is not abundant^31^. As a result, the greatest obstacle to collecting HM samples in the present cohort was represented by the difficulty in establishing milk supply, which underpinned nearly half of the missed samples (Fig. 3), despite all the recruiting units adhering to the baby friendly initiative. Establishing milk supply soon after birth is a challenge faced by most mothers who give birth prematurely, making preterm birth a risk factor for inadequate HM supply (52% of preterm mothers versus 17% of term mothers)^32^. Furthermore, delayed onset of lactogenesis has often been associated with several maternal factors, including stress^33^, obesity and diabetes^34^, caesarean section^35^ and exposure to antenatal glucocorticoids^36^. However, none of these factors showed an association with the volumes of HM expressed during collection in our cohort. This may be due to the fact that HM volumes for the present study were representative of a single expression and not of the 24 h period or to the small sample size of specific subgroups. Nonetheless, differences in HM production were related to maternal ethnicity, with Asian mothers producing the least and Pacific Islander mothers producing the most HM on day 5. Interestingly while previous research has not reported correlations between maternal ethnicity and HM volumes^37^, other studies do suggest that ethnicity may be a predictor of lactation outcomes, especially in association with BMI and/or gestational weight gain^38^. Maternal education was also associated with expressed HM volumes in our cohort, but surprisingly in contrast with previous studies^37^ mothers with the highest degree were observed to express the lowest volume on the day 5 collection. However, for both the findings relative to maternal ethnicity and those relative to maternal education we did not have the possibility to verify the impact of parity, which could have played an important role in both cases (e.g. Pacific islanders might have had previous infants which would have helped with HM production and vice-versa for mothers with high education this could have been the first pregnancy exposing them to higher risk of lower HM supply^39^).

Limitations of the present study include the fact that the research was retropsectivly designed, thus lacking the means to collect further data that could affect HM supply (e.g. use of medications to improve lactogenesis) and collection (e.g. acceptability of HM collection from clinical staff in each unit) or to accurately investigate the nature of the observed challenges (e.g. lack of lactation support to mothers^40,41^). For instance we only managed to record the reason for missed sample collection and did not have consistent data on the reasons around deviation from the collection protocol (i.e. why did mothers not empty the breast 2–3 hours before collection, why were the samples collected out of the recommended timing). The protocol change from variation A to B had to be put in place due to the fact that some mothers did find a HM collection on day 3 overwhelming. The small sample size, may have failed in detecting clinically significant differences across subgroups in regards to HM volumes. Moreover, because of the multiple centers involved in the trial, sample collection was performed by different people who had their own approach to mothers, this most likely impacted the adherence to the standardized sampling protocol. Finally while we tried to standardize sample collection, it is necessary to take into consideration that the nature of the sample constitutes a standalone limitation. In fact while we collected samples from the right breast for all mothers, some mothers present asymmetrical production of HM^40^, which impacts the reported volumes when only one breast is used to express^42^.

To our knowledge, this is the first study exploring the feasibility to HM collection for research purposes in NCU. Our results highlight that, while achievable, HM collection in NCUs presents difficulties related to the standardization of collection conditions, at least in the targeted population. Establishing specific rules to contact mothers and schedule the collection time as well as ensuring that mothers understand the importance of the standardized collection procedure may improve adherence to the sampling protocol. Furthermore making sure that mothers understand the importance of the standardized procedure is also necessary. Finally in a clinical environment such as NCUs, where clinical staff are concerned about the immediate health issues of mothers and infants and HM research is not a priority, having effective communication between researchers and practitioners is crucial to ensure the success of sample collection. For this reason, the acceptability of HM sampling for research purposes, which was not assessed in this instance, should be investigated, both amongst mothers and clinical practitioners. This will aid in targeting possible internal barriers to accurate data and sample collection, ultimately improving the quality of HM research.

## Data Availability

The dataset generated and analysed during the present study is available from the corresponding author on reasonable request.
